# Efficacy and safety of colchicine in the treatment of acute myocardial infarction: A protocol for systematic review and meta-analysis

**DOI:** 10.1097/MD.0000000000025429

**Published:** 2021-04-09

**Authors:** Hui Xiong, Xianli Huang, Lingzhang Rao, Jinhe Zhao

**Affiliations:** a Department of Cardiology, Wuhan Wuchang Hospital, Wuchang Hospital Affiliated to Wuhan University of Science and Technology; b Department of Cardiology, Tianyou Hospital Affiliated to Wuhan University of Science and Technology, Hubei, China.

**Keywords:** acute myocardial infarction, colchicine, meta-analysis, protocol, review

## Abstract

**Background:**

There are no meta-analyses evaluating the efficacy and safety of colchicine in the treatment of acute myocardial infarction (AMI). Our protocol is conceived to evaluate the efficacy and safety of colchicine in comparison of placebo and test the hypothesis that a short course of treatment with colchicine could lead to reduced infarct size in patients presenting with AMI.

**Methods:**

We will follow the Preferred Reporting Items for Systematic Reviews and Meta-Analyses (PRISMA) reporting guidelines and the recommendations of the Cochrane Collaboration to conduct this meta-analysis. Reviewers will search the PubMed, Cochrane Library, Web of Science, and EMBASE online databases for all English-language cohort studies published up to April, 2021. The cohort studies focusing on assess the efficacy and safety of colchicine in the treatment of AMI will be included in our meta-analysis. At least one of the following outcomes should have been measured: reduced infarct size, C-reactive protein (CRP) level, adverse events, death and major cardiovascular events. Review Manager software will be used for the meta-analysis. All outcomes are pooled on random-effect model. A *P* value of <.05 is considered to be statistically significant.

**Results:**

Our protocol is conceived to evaluate the efficacy and safety of colchicine in comparison of placebo and test the hypothesis that a short course of treatment with colchicine could lead to reduced infarct size in patients presenting with AMI.

**Registration number:**

10.17605/OSF.IO/NTU5F.

## 1 Introduction

Inflammation is common after acute myocardial infarction (AMI). The massive loss of myocardium through cell necrosis, apoptosis, and autophagy activates a strong inflammatory response that develops due to recruitment of inflammatory cells and induction of the expression of inflammatory cytokines and chemokines.^[1–3]^ The most commonly used circulating biomarker clinically is C-reactive protein (CRP), with a peak frequently observed around day 3 after AMI. In the case of AMI, inflammation is closely associated with the pathophysiology of ischemia-reperfusion injury and fibrosis.^[4,5]^ As mentioned previously, inflammation plays a deleterious role at the onset of reperfusion. It can lead to infarct size and the process of heart remodeling, which can lead to heart failure.^[6]^ Inflammatory status is a major predictor of adverse events after AMI. Therefore, inflammation seems to be a promising therapeutic target for patients with AMI. Several anti-inflammatory therapies appear to be potential candidates, but so far, there has been little research in this area.^[7,8]^

Colchicine is a well-established anti-inflammatory drug that is commonly used to treat gout, and it also targets the inflammasome. Colchicine is also used as a treatment for pericarditis. It may exert multipotent anti-inflammatory effects, particularly by inhibiting neutrophils migration, and may also have direct anti-inflammatory effects by inhibiting key inflammatory signaling networks called inflammasomes and proinflammatory cytokines.^[8,9]^ In addition, colchicine has been shown to have anti-atherosclerosis effects and has been proposed to reduce inflammation in patients with stable coronary heart disease.^[10]^ Recently, colchicine has been shown to decrease infarct size, with a reduction in the concentration of creatine kinase muscle-brain fraction and infarct size on cardiac magnetic resonance imaging in patients with AMI.^[11,12]^

Currently, there are no meta-analyses evaluating the efficacy and safety of colchicine in the treatment of AMI. Our protocol is conceived to evaluate the efficacy and safety of colchicine in comparison of placebo and test the hypothesis that a short course of treatment with colchicine could lead to reduced infarct size in patients presenting with AMI.

## 2 Materials and methods

### 2.1 Search strategy

**Figure 1:**
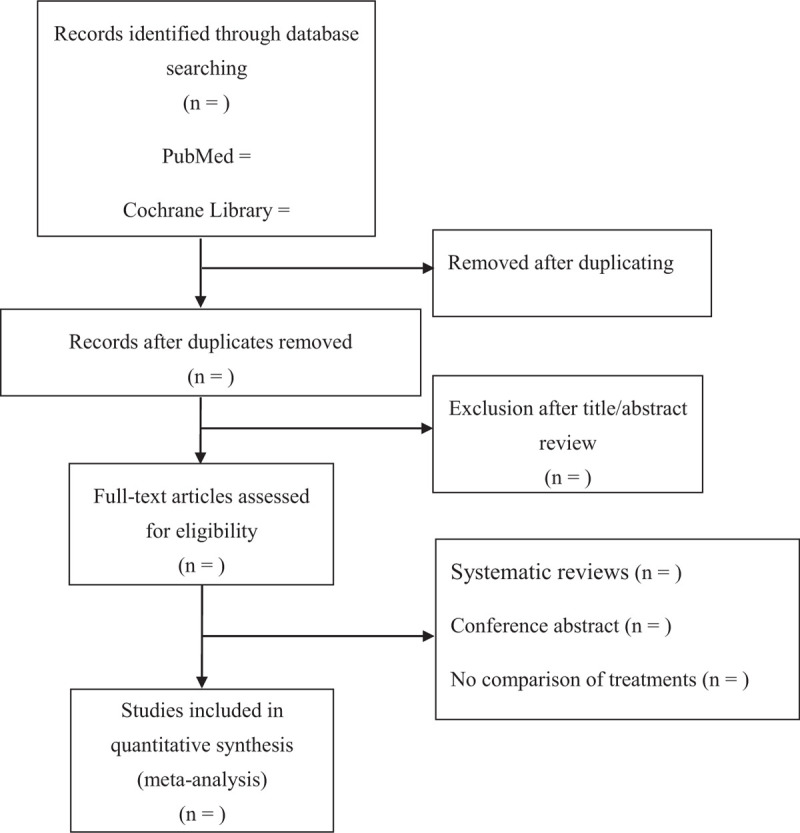
Flow diagram of study identification.

We will follow the Preferred Reporting Items for Systematic Reviews and Meta-Analyses (PRISMA) reporting guidelines and the recommendations of the Cochrane Collaboration to conduct this meta-analysis. The detailed guidelines can be found at www.prisma-statement.org. The systematic review protocol has been registered on Open Science Framework registries (https://osf.io/ntu5f). The registration number is 10.17605/OSF.IO/NTU5F. Reviewers will search the PubMed, Cochrane Library, Web of Science, and EMBASE online databases using the key phrases “colchicine,” “acute myocardial infarction,” and “AMI” for all English-language cohort studies published up to April, 2021. Ethical approval is not necessary because the present meta-analysis will be performed based on previous published studies. Flow diagram of study identification is shown in Figure 1.

### 2.2 Eligibility criteria

The cohort studies focusing on assess the efficacy and safety of colchicine in the treatment of AMI will be included in our meta-analysis. At least one of the following outcomes should have been measured: reduced infarct size, CRP level, adverse events, death and major cardiovascular events. The exclusion criteria contain biochemical trials, reviews, case reports, no assessment of outcomes mentioned above, and no comparison of colchicine and placebo.

### 2.3 Data extraction

In order to achieve a consistency (at least 80%) of extracted items, the data extractors will extract data from a sample of eligible studies. Results of the pilot extraction will be discussed among review authors and extractors. Two independent reviewers will extract data with a predefined extraction template, which includes the following items: study characteristics such as the first author, publication year, study design, follow-up period; patient demographic details such as patients’ number, average age, and gender ratio. The outcomes include reduced infarct size, CRP level, adverse events, death and major cardiovascular events. The original authors will be contacted to request missing data where necessary. Extracted information will be cross-checked by 2 independent reviewers. Any disagreements will be discussed and resolved in discussion with a third reviewer.

### 2.4 Data analysis

Review Manager software (v 5.4; Cochrane Collaboration) will be used for the meta-analysis. Continuous variables are extracted and analyzed to mean value ± SD. Standardized mean differences with a 95% confidence interval are assessed for continuous outcomes. The heterogeneity is assessed by using the Q test and *I*^*2*^ statistic. An *I*^*2*^ value of <25% is chosen to represent low heterogeneity and an *I*^*2*^ value of >75% to indicate high heterogeneity. All outcomes are pooled on random-effect model. A *P* value of <.05 is considered to be statistically significant.

### 2.5 Assessment of methodological quality

In order to achieve a consistency (at least 80%) of risk of bias assessment, the risk of bias assessors will pre-assess a sample of eligible studies. Results of the pilot risk of bias will be discussed among review authors and assessors. Two independent reviewers will assess the risk of bias of the included studies at study level. We will follow the guidance in the latest version of Cochrane Handbook for systematic reviews of interventions when choosing and using tools to assessing risk of bias for randomized trials (version 2 of the Cochrane risk of bias tool for randomized trials, RoB 2) and non-randomized trials (the Risk of Bias in Non-randomized Studies of Interventions, ROBINS-I tool). Any disagreements will be discussed and resolved in discussion with a third reviewer. Studies with high risk of bias or unclear bias will be given less weight in our data synthesis.

## 3 Discussion

In AMI, acute ischemia causes myocardial necrosis with subsequent endogenous inflammation, leading to myocardial damage, ventricular dilation, and dysfunction. Recently, colchicine has been shown to decrease infarct size, with a reduction in the concentration of creatine kinase muscle-brain fraction and infarct size on cardiac magnetic resonance imaging in patients with AMI.^[11,12]^ Currently, there are no meta-analyses evaluating the efficacy and safety of colchicine in the treatment of AMI. Our protocol is conceived to evaluate the efficacy and safety of colchicine in comparison of placebo and test the hypothesis that a short course of treatment with colchicine could lead to reduced infarct size in patients presenting with AMI.

## Author contributions

**Conceptualization:** Lingzhang Rao.

**Data curation:** Hui Xiong, Xianli Huang.

**Formal analysis:** Hui Xiong, Xianli Huang.

**Funding acquisition:** Jinhe Zhao.

**Investigation:** Hui Xiong, Xianli Huang.

**Methodology:** Lingzhang Rao.

**Project administration:** Jinhe Zhao.

**Resources:** Lingzhang Rao, Jinhe Zhao.

**Supervision:** Jinhe Zhao.

**Validation:** Lingzhang Rao.

**Writing – original draft:** Hui Xiong, Xianli Huang.

**Writing – review & editing:** Jinhe Zhao.
